# Supplementary material for: A single touch can provide sufficient mechanical stimulation to trigger Venus flytrap closure
Source: PLoS Biol. 2020 Jul 10;18(7):e3000740. doi: 10.1371/journal.pbio.3000740 (PMC7351144; doi:10.1371/journal.pbio.3000740)
Supplement: Attachment — Submitted filename: ResponseToReviewers.docx [file pbio.3000740.s008.docx]

Table of Contents

[Detailed Response to the Reviewers’ Comments	1](#_Toc32227218)

[Reviewer 1	1](#_Toc32227219)

[Reviewer 2	9](#_Toc32227220)

## Detailed Response to the Reviewers’ Comments

First, we would like to thank the reviewers for their insightful and constructive criticisms. We have tried to address all the points they made in the revised version of our manuscript. Below you can find a point-by-point response to the reviewers’ comments. For ease of reading, the reviewers’ comments are shown in blue and our responses in italics.

In addition to the requested changes, we replaced a reference, Volkov et al., 2011, J Plant Physiol. (reference 26 in the original manuscript) with Volkov et al., 2008, Plant Physiol. (ref. 32 in the revised manuscript) because it more specifically discusses the trap closure mechanism and is better suited to support our statements. Since our first submission to PLOS Biology the affiliation of Hans J. Herrmann has changed, which we adapted in the manuscript. We also added an additional affiliation for Ingo Burgert and Markus Rüggeberg.

### Reviewer 1

This is a highly interesting study of how the Venus flytrap closure is triggered. Is main strength is the simultaneous measurement of the force applied to the sensory hair, of the electric potential, and of the force associated with trap closure. However, the study and the writing suffer from a number of weaknesses. The data could be better used and the presentation could be greatly improved.

We thank reviewer 1 for this positive and helpful review. He/she pointed out a few shortcomings, which greatly helped us to improve the manuscript.

MAJOR

1) The authors make the implicit assumptions that sensing occurs at the base of the hair, that the hair remain straight, and that angular velocities at the base can be computed using displacement and distance from base. What is the evidence that angles are more relevant? (For instance, experiments on the same hair at different distances from the base.) How straight are hairs during force application? (One of the movies gives the impression that the hair bends.)

Using angles allows us to normalize for different contact heights and different sensory hair geometries. We have good evidence that the sensory hair bends mainly at the constriction, whereas the shaft is hardly deformed. We added movies for the reviewers (i.e., sideview_bend.avi and new Video S2 in the revised manuscript).

2) There is some inconsistency about what is sensed. It is stated based on preliminary experiments that the flytrap is sensitive to angular velocity, whereas Fig 1E implies that a total angular displacement is sensed. The authors could make a better use of their data on closure triggered by single deflection to check whether closure is better predicted by cumulated angle, by cumulated absolute velocity, or by another combined quantity.

We thank Reviewer 1 to point out these ambiguities. In the manuscript, we state that static displacement does not contribute to the receptor potential (Fig. 1 F) whereas the rate of displacement, or a change in displacement, does. At the high velocities used for the double deflection experiments, the duration of the deflection is much shorter than the time-dependent factors, such as the decay of the receptor potential (Benolken and Jacobson, 1970, The Journal of General Physiology) and relaxation of the hair (Figure 1 F). Therefore, we assumed that in this case, a deflection can be approximated by a discrete increase of angular displacement from zero to the total value and, as a consequence, the firing of an action potential (AP) depends mostly on the total angular displacement. Furthermore, the speed of deflection in our experiment is in a similar range as the movement of classical prey, also confirmed by recently published data (Scherzer et al., 2019, Nature Plants; Balebail et al., 2019, PLOS ONE; for more details see “MINOR point 3”). Thus, we used our data to find the minimal angular deflection a prey would need to apply to generate APs and trigger trap closure (Fig. 1 C and D). Figure 1 E is a visualization of this angular displacement threshold, showing that below 0.12 rad we never saw trap closure (the equivalent is valid for the torque). We now explain the reasoning for using a displacement threshold in the manuscript and explicitly mention the lower boundary in the text. Interestingly, the influence of the angular velocity becomes much more prominent for single deflection trap closure, which occurs at lower angular velocities. The ability to elicit two APs with one deflection clearly depends on a combination between angular velocity and angular deflection. To study the role of the velocity, we performed experiments where we kept the displacement of the respective hair as constant as possible and changed the velocity (Fig. 3 A). Using this approach, we showed that single deflection triggering only occurs in a certain velocity range (for sufficiently large deflections, Fig. 3 B). Of course, even within this velocity range, the angular displacement (or the duration of the deflection) must be large enough to cause two APs. Plotting all the single deflection data in a parameter space (angular displacement vs. angular velocity), we can define a region in which the combination of velocity and displacement leads to single deflection closure (Fig. 3 B). This region consists of a lower and upper velocity boundary, and a lower displacement boundary that is similar to the region predicted by the model.

3) What are the variations with velocity/angle of the delay between APs and closure? Is this predicted by the model?

The trap always closes in response to a second AP. An AP is an all-or-nothing response (switch), and should always look the same (a discrete value), independent of the magnitude of the stimulus. Therefore, the closure should be decoupled from the receptor potential and the type of the mechanical stimulus preceding the second AP, and the delay between AP and closure should be the same. However, we don’t have the necessary time resolution to rule out that the delay time is affected.

4) If mechanosensitive channels are involved, how do the authors explain that both positive and negative angular velocity contribute to closing? This should be a discussion point.

When the trigger hair is deflected, the membrane is compressed on one side and stretched on the other, thought to open the mechanosensitive channels. The membrane acts like a fluid and has a tendency to relax, which closes the channels again. That is also why sustained deflection does not lead to trap closure. When the trigger hair moves back into its original position, the membrane is stretched/compressed on the opposite side.

5) What is the precision of the different measurements?

We do not understand what exactly the reviewer means but the specifications (resolution) of the system are given in the Materials and Methods section. If the reviewer is referring to error bars, every data point is an individual measurement as the trap eventually closes.

6) What is the justification for the forms of the equations used in the model? (Types of functions chosen, etc.)

We use four core equations and tried to use the mathematical form of equations that have the least number of parameters but still satisfy the respective requirements:

a) The charge buildup equation (6): The core of the charge buildup equation was empirically developed from the observed behavior of experiments and by reasoning. After identifying the relevant property (angular velocity ($\omega$)) we took the simplest form—a linear relation—with a constant rate factor $k$. However, it needs some refinement to meet experimental observations on the desensitization effect that is highlighted by Scherzer and colleagues (ref. 20 in the revised manuscript). A charge feedback is sought to represent a situation where the response to the stimuli gets progressively weaker, meaning that there is a gradual decrease in the charge buildup ($\Delta Q_{RP}^{n+1}$) due to an increase in previously accumulated charge ($Q_{RP}^{n}$) up to the previous step. In other words, the rate factor $k$ needs to be reduced in one way or the other. The choice of that function (term in brackets of Eq. 6) is arbitrary and we originally used a 3-parameter continuous decay function. However, we realized that a sigmoid function that asymptotically goes from value $1$ (when $Q_{RP}=0$) down to the asymptote at $0$ (for $Q_{RP}\to\infty$), like the sigmoidal membership function, only needs 2 parameters and is thus preferable. Unfortunately, the experimental basis for a sound choice of the parameters $a,c$ is not available; hence, we had to identify these parameters inversely. The passage in the manuscript now reads:

$$\Delta Q_{RP}^{n+1}=k\cdot\left( 1-\frac{1}{\text{exp}(-a(Q_{RP}^{n}-c))} \right)\cdot\omega(t)\cdot\Delta t.$$

“The sigmoidal membership function provides a continuous desensitization for increasing $Q_{RP}^{n}$ with the parameters $a=1.99,c=3.48$, while $k$ is obtained as $k=40.2\mu C/rad$ to best describe the experimental results.”
b) The flux limitation law (7): A model also has to give a reasonable model response for the entire range of input parameters. Infinite charge accumulation has to be restricted for high velocities (beyond typical deflection rates) because electrical memory in tissues would have a physical limit to store charge. We could have taken a discrete maximum value. As such a switching behavior would be quite strange for a biological system, and also to avoid discontinuities, we used a simple sigmoid function (the logistic function) that converges to the maximum flux ($\dot{Q}_{RP}^{lim}$).
c) The exponential receptor potential decay law (8): The exponential decay is based on the description in Volkov et al., 2009, Plant Physiol. (ref. 33 in the revised manuscript), where Volkov proposed an equivalent electrical circuit for the Venus flytrap.
d) The exponential short term memory decay law: same as c)/equation (8).
7) How were model parameters chosen? Is it eye-balling or was a systematic fitting procedure applied (the former is not acceptable). How sensitive are the results to parameter values?

We thank the reviewer for her/his comments towards the proposed model. We originally did not look at the sensitivity of the results; however, the reviewer is correct to point out the importance of a sensitivity analysis for such ill-defined problems. We are fully aware that we are dealing with a phenomenological model for reasons that are rooted in the nature of a living biological system with significant variability and scarce observations. Like all models, it is for sure not correct in all aspects—there is most likely no unique parameter set that would perfectly represent the limited (and not always consistent) experimental observations—and it is based on multiple assumptions and simplifications, pointed out throughout the manuscript. However, it is useful to understand how experimental parameters and observations are related in order to distinguish important from less important parameters, and to complement the picture beyond the scarce experimental observations.

For the parameter identification, we combined pre-existing knowledge on the range of certain parameter values, with stepwise iteration. The model was constructed by analytical expressions based on general principles mentioned in the literature. As a step by step procedure, we based our model on these known parameters ($\tau_{RP},\tau_{STM},t_{RP},Q_{STM}^{th},Q_{RP}^{th}$) to define the founding equations and, iteratively, we incorporated tuning parameters ($a,c,k$) for our mathematical functions, such that they reproduce the situation in Fig. 3 C.

As a summary, below are the lists of parameters and the reasoning behind their selection. Note that this is all described in the manuscript at one place or the other. We consider the parameter choice as sufficiently good when the AP firing time and angle measurements of Fig. 3 C are correctly predicted AND the largest possible fraction of single deflection triggering experiments (Fig. 3 B) and all double deflection triggering experiments (Fig. 1 D) lie in the respective phase space AND the largest possible fraction of double deflection triggering experiments only fire 2 APs. The fitting is a multi-dimensional optimization procedure that minimizes the multi-objective function (3 objectives) that consists of the sum of (a) the portion of falsely predicted realizations and (b) the mean square error of the predicted AP triggering time differences (Fig. 3 C) weighted by a factor of 10 and (c) the portion of wrongly predicted number of fired APs for double deflection triggering experiments. Since these technicalities go way beyond a biology journal, we did not include it. We estimated the parameter set of the 7 free parameters (see below), by a surrogate model, using the MATSuMoTo Matlab toolbox by Juliane Müller (Müller et al., 2013, Computers and Operations Research). The initial design, meaning the starting point distribution in the 7-dimensional parameter space was Latin hypercube sampling, the surrogate model is based on cubic radial basis functions, and the sampling strategy combines local candidate point search with the global, to avoid being locked in local minima. Note that in the previous version, we used as experimental reference the average of the last non-triggered parameter and the triggered one. We realized that this “estimate” for the phase boundary was not well defined, since it depended on the increment of the tested parameters. As a result, we obtained extremely high characteristic decay constants for the sensory memory, which in consequence triggered too many APs for large deflections. We corrected this and now only use experimental points that really triggered. The resulting optimal parameter set is given in the table below:

Fixed parameters:

| ***Symbol*** | ***Value*** | ***Description*** *(reference numbers according to the revised manuscript)* |
| --- | --- | --- |
| $Q_{STM}^{th}$ | *10 µC* | *Threshold charge accumulation in the Short Term Memory needed to initiate trap closure. Volkov suggests in [26] that the threshold is 8 µC for small traps and 9 µC for larger traps* |
| $Q_{RP}^{th}$ | *1 µC* | *Charge threshold corresponding to the Receptor Potential (RP) threshold, necessary to trigger an AP. This value is lower than the total charge required for trap closure.* |
| $Q_{AP}$ | *6 µC* | *Amount of charge transferred from SM to STM after an AP.* |

Free parameters:

| ***Symbol*** | ***Value*** | ***Description*** *(reference numbers according to the revised manuscript)* | ***PCE Sobol 1*** | ***LRA Sobol 1*** |
| --- | --- | --- | --- | --- |
| $\tau_{RP}$ | *0.64 s* | *Characteristic decay constant to represent Sensory Memory (SM). Its value was suggested by Volkov in [33] to range between 0.3 and 3 s* | *0.002506* | *0.022990* |
| $\tau_{STM}$ | *39.7 s* | *Characteristic decay constant to represent Short Term Memory (STM). Its value was suggested by Volkov in [33] to range between few seconds to a minute.* | *0.0* | *0.000147* |
| $t_{RP}$ | *1.03 s* | *Refractory period between two Action Potentials (APs). It is found in plants to have an average value of 0.75 s [28]* | *0.029770* | *0.008877* |
| $\dot{Q}_{RP}^{\text{lim}}$ | *93.2 µC/s* | *Maximum charge flux. This value has to be chosen such, that the snapping phase diagram is limited for high angular rates.* | *0* | *0.016855* |
| $k$ | *40.2 µC/rad* | *Sensitivity multiplier relating* $Q_{RP}^{n+1}$ *to angular velocity (ω)* | *0.869190* | *0.892449* |
| $a$ | *1.99* | *Tuning function parameter to fit results in Fig. 3 C* | *0.000000* | *0.002845* |
| $b$ | *3.48* | *Tuning function parameter to fit results in Fig. 3 C* | *0.000000* | *0.009241* |

To clarify this we added the following sentence to the manuscript:

“Note that $Q_{STM}^{th},Q_{RP}^{th}$ and $Q_{AP}$ are fixed a priori, while the other parameters ($\tau_{RP,STM},t_{RP},\dot{Q}_{RP}^{lim},k,a,c$) are determined by a multi-dimensional optimization procedure using surrogate models [34]. The ideal parameter set is reached when (1) all experiments are correctly predicted, (2) the AP triggering sequence of Fig. 3 C is correctly calculated, (3) the maximum of double deflection experiments trigger 2 APs, and (4) parameters are within reasonable physical and biological bounds.”

We also updated all values to the parameter set obtained with the simplified desensitization function by the optimization procedure and added a reference (Müller et al., 2013, Comput Oper Res, which is ref. 34 in the revised manuscript).

The sensitivity of the parameters is not so easy to quantify, as we can only speculate on the statistical distribution of the parameter values in the biological system. To quantify the sensitivity of the involved parameters, we calculated the Sobol indices for the objective function used in the parameter identification. The Sobol indices can be calculated from Monte Carlo (MC) sampling or via meta-models. Since one evaluation of the objective function takes about 120 s in our implementation, MC sampling is barely possible (~3.8 years for 1e6 samples). To create the meta-model, we employed polynomial chaos expansion (PCE) and, for comparison, a canonical low-rank approximation (LRA) meta-model within the free UQlab (uncertainty quantification toolbox v. 1.3 www.uqlab.com) by ETH Zurich (Chair of Risk, Safety and Uncertainty). We start from the identified, optimal parameter set and employ normal distributions with a standard deviation of 0.1 times the respective mean value. In the table, we show the first order Sobol indices (estimated after 200 variations) that evaluate the importance of one variable at a time. We can see the dominating importance of the sensitivity multiplier $k$ emerging in both meta-models, meaning that more than 85% of the variance of the model accuracy (objective function) is caused by the variance of $k$. Different assumptions of the input parameter disorder will result in different estimates; however, the dominance of $k$ will remain. To include this important insight into the manuscript we added the sentence:

“Using polynomial chaos expansion we calculated the relative importance of the variance of each model input parameter for the variance of the model prediction accuracy. The resulting Sobol indices revealed a dominating influence of the sensitivity parameter $k.$”

MINOR

1) Acronyms are overused.

We do not understand this comment as we use only two acronyms (RP and AP), which are applied according to the author guidelines of this journal.

2) Captions are not always sufficiently detailed.

We thank the reviewer for this comment. Captions were extended where necessary.

3) How comparable are the operating velocity and natural velocities associated with preys?

According to Scherzer et al., 2019 (Ref. 20 in the revised manuscript), ants deflect the sensory hair with an angular velocity between 14.5 and 448°/s, which corresponds to 0.25-7.8 rad/s. This is in the same order of magnitude as our operational speed, which was between 10 and 20 rad/s for the double deflection experiments and between 0.01 and 4 rad/s for the single deflection experiments. According to the high-speed recordings made available in the supplementary data, as well as personal feedback provided by Balebail et al. (Balebail et al., 2019, PLOS ONE), the linear leg velocity of typical prey, e.g. houseflies, are in the range of 1.5 m/s, which is even higher than the linear motion of our force sensor during the fast double deflection experiments. We added these examples and the corresponding references to the manuscript (Balebail, 2019, PLOS One is ref. 22 in the revised manuscript).

4) Figure 2B is confusing. Perhaps specify “Number of deflections to trigger closure”.

The figure title was changed according to Reviewer 1’s suggestion. In addition, we changed the title of Fig. 2 A to "Number of triggered APs per deflection."

### Reviewer 2

The authors study the touch sensitivity of the carnivorous Venus flytrap in the context of animal prey recognition. This is a topic of great interest to the reader of PLOS Biology. The authors have developed an electromechanical model from which they suggest that, under certain circumstances, one touch is sufficient to generate two action potentials. This model challenges findings well documented by other labs that two APs are required to close the trap. Do the author’s experiments support their hypothesis?

We thank Reviewer 2 for his/her extensive review of our manuscript. In contrast to what he/she states in the first sentence, however, we did not study the Venus flytrap in the context of animal prey recognition. More accurately, we used a completely artificial triggering system to study the trap closure mechanism from an electromechanical point of view under controlled conditions on living and intact Venus flytraps. This allowed us to define the parameter spaces which are able to elicit APs and lead to trap closure. The main new finding was that a range of parameters exist that allow trap closure after a single deflection. However, we do not disagree with the well documented fact that two APs are necessary for trap closure. Our experiments as well as our mathematical model clearly demonstrate that 2 APs have to be elicited in a single deflection to trigger trap closure.

General comment:

A recent Scientific Reports paper by Poppinga et al. discussing the two trigger hair biomechanics and -electrics papers published

Scherzer, S. et al. Venus flytrap trigger hairs are micronewton mechano-sensors that can detect small insect prey. Nat Plants. 5, 670-675 (2019).

and the bioRxiv version

Burri, J. T. et al. The mechanical basis for snapping of the Venus flytrap, Darwin’s ‘most wonderful plant in the world’. bioRxiv. <https://doi.org/10.1101/697797> (2019).

of the PLOS Biology paper under discussion side by side.

Why the authors did not discuss the findings of Scherzer et al. 2019 publicly available since some time?

We submitted our manuscript to bioRxiv at the same time the study by Scherzer and colleagues was published and transferred this version to PLOS Biology. We have now extended the discussion of Scherzer et al. 2019.

Questions/Answers:

Authors: Our results confirm the model’s predictions, suggesting that the Venus flytrap may be adapted to a wider range of prey movement than previously assumed.

Question: What is the evolutionary advantage of a system able to respond to a single touch that is equivalent to background noise?

Comment: The authors have not addressed the fact that Dionaea can catch animals of different size from large and small traps, with the latter growing trigger hairs that are more sensitive to force (Ref 18).

Single deflection triggers occur only under relatively slow angular velocity conditions, much slower than those generated for example by a drop of water or a piece of dead organic matter falling onto the trap. As we state in our manuscript, we do not know for sure whether there is a biological function behind this mechanism and can only speculate about it and suggest that the Venus flytrap may also capture slow moving prey such as snails or larvae. The fact that traps of different sizes have trigger hairs with different sensitivities does not relate to our statement that is questioned here by Reviewer 2.

Authors: The mechanical basis for snapping of the Venus flytrap, Darwin’s `most wonderful plant in the world’

Comment: Why is Darwin’s statement in the title and first sentence of the introduction in a paper on biomechanics and electrics of the trigger hair that very likely is not understood by general readers of PLOS Biology?

Like Reviewer 2 in his introduction, we think that this article is of great interest to the readers of PLOS Biology because we present a reconsideration of the electromechanical basics of the wonderful catching mechanism of the Venus flytrap. In contrast to Reviewer 2, however, we think that the general reader of PLOS Biology is by all means capable of understanding the principles described here, and we do our best to support them.

Authors: .. starting with the observations made by Moses A. Curtis in the 1830s [2].

Comment: The observations that Moses A. Curtis made should be stated clearly.

We extended the sentence to specify Curtis’ conclusions. The observations themselves can be found in the cited reference (Curtis, 1834, Boston J. Nat. Hist.). We also added a sentence and two new references to corroborate the importance of Curtis’ insight (Edwards, 1804, Curtis’s Botanical Magazine; Nuttall, 1818. Refs. 2 and 3 in the revised manuscript).

Authors: Previous attempts to correlate the mechanical stimuli to the generation of APs … were not quantitative, or the experiments were done in vitro using dissected traps [18].

Comment: When reading Ref 18, this statement appears incorrect and misleading. In contrast to the authors’ statement, experiments in Ref 18 were conducted with intact Dionaea plants.

We are wondering where the reviewer found hints for the use of intact plants/traps in any of the experiments. Unfortunately, Scherzer and colleagues are not giving an overview of the setup used for the trigger hair deflection and AP measurement experiments. Below, we detail why we concluded that Scherzer and colleagues did not use intact traps. Citations from Scherzer et al. are indicated by quotation marks.

Tissue stress experiments:

“Venus flytraps were **bisected** along the midrib and fixed with double-sided tape.”

Venus flytrap hair analysis:

1. Ant experiment:

- “To observe the mechanical stimulation of Dionaea trigger hairs under **seminatural** conditions, we filmed the surfaces of three snap-traps, each containing **three trigger hairs**, …”
- An entire trap contains ≥ 6 trigger hairs.
- “… snap-trap **mounted** on its upper side using double-sided tape, …”
- Fig. S1 and Supplementary Video 3 clearly show that the traps were detached from the plant, bisected, and mounted to the supporting bridge.

2. Trigger hair deflections using the force transducer

- The only hint on the trap state comes from Supplementary Video 4, which looks like the trap was bisected or unnaturally twisted to create space for the force transducer (see direction of the long “teeth” on the edge of the trap). Both situations can hardly be called intact plants/traps.

3. Temperature dependence of APs experiment

- “… was analyzed by **glueing a flytrap lobe** with its outer surface on a glass cylinder …”
- Again, this is a highly artificial situation where the inner trap lobe is bent into a highly convex shape to expose the sensory hair (which seems to be the case for the experiment presented in Supplementary Video 4).

For these reasons, we think that the statement which is questioned by the reviewer is correct

Authors: To overcome these shortcomings, we used a microelectromechanical (MEMS)-based force sensor mounted on a microrobotics system to precisely control the velocity and amplitude of the deflection and to simultaneously measure the applied force in vivo

Comment: Why was it necessary to measure the snap force and repeat experiments performed by Volkov et al., 2012? What extra information could be gained compared to a simple video observation of the closing movement?

Most importantly, the experimental setup used to measure snap force has likely interfered with the recording of surface action potentials, because two metallic arms were in direct contact with the inner surface of both trap lobes (line 251). The tissue of the inner trap lobe is electrically excitable and action potentials can be generated just by mechanical stimulation of its surface. Thus, any small movements of the trap relative to the metallic levers (e.g. due to vibrations, particularly during the high accelerations involved in the piezo movements) can stimulate the trap (at a different location), leading to complex artefacts.

As far as we are aware, the trap closing force data presented by Volkov and colleagues (2012) are the only ones available to date. We think that there is nothing wrong in repeating experiments done by others. We even consider it necessary to confirm (or contradict) results that have not been validated, if possible by using a different method. This is the only way to obtain scientific, reliable information. Therefore, our contribution is at least a confirmation of Volkov’s results. We added another reference (Volkov, 2012, J Plant Physiol; ref. 29 in the revised manuscript), which we overlooked before. Also, from a simple video observation as suggested by Reviewer 2, it would be extremely difficult to extract force information.

Interference with AP recording using our setup was one of our initial concerns. However, we saw that this setup did not trigger unwanted trap movements and/or APs during trigger hair deflection by the force probe. One of the functions of the two metallic arms was to protect the MEMS force sensor from damage by snapping traps. Therefore, for many of the experiments, the metallic arms were not in direct contact with the trap lobes, rather the contact was made by the conductive gel we used.

Scherzer and colleagues used the same method, although they measured the surface potential on the outer trap lobe. Since the outer trap lobe also reacts to mechanical stimulation, which leads to closure, measuring the surface potential of the inner lobe is equally valid. Furthermore, we confirm that the system does not move apart from the MEMS sensor in contact with the trigger hair (see new Video S2 in the revised manuscript and sideview_bend.avi).

Fast movements of the MEMS sensor would produce stronger vibrations. If this had been a problem, fast movements would generate several APs, which is clearly not the case (see data presented in the article). When the trap closes, we get high background noise, which is indicated in Figs. 3 C and D

Authors: Reasoning that hair deflection induced by spiders, ants, and flies, i.e. the ‘classical’ prey of D. muscipula [19], would be rather quick, we operated the microrobotics system at full speed to simulate these stimuli, resulting in high initial angular velocities ranging from 10 to 20 rad s-1.

Question: To understand the authors’ reasoning, readers need to know how fast typical Dionaea prey move and how far trigger hairs get bent upon collision with typical prey animals. The angular velocities used here are actually higher than the range observed for deflections of running ants in Ref 18 (14.5 to 448 °/s, corresponding to 0.25-7.8 rad/s).

The angular velocities we used were in the same order of magnitude as those presented by Scherzer and colleagues in the running ants experiment, which confirms that our assumption was correct. Houseflies even move their legs at a speed of about 1.5 m/s, which corresponds to an angular velocity of >750 rad/s (our own video analysis on data from Balebail et al., 2019, PLOS ONE). We added these examples and the corresponding references to the manuscript (new ref. 22 in the revised manuscript: Balebail, 2019, PLOS One).

Authors: If the trap did not close, we waited for 2 min to make sure that the RP was completely reset.

Comment: Surface electrodes in general and those the authors used in particular do not monitor the receptor potential, not even the membrane potential (Volkov, 2018).

Indeed, we have not measured the RP and never claimed to have done so. What we measure is the AP, which works perfectly well with the surface electrodes we used, as our data show. The reason for choosing a 2 min waiting time is based on well-established data from the literature, which states that the second AP needs to be elicited within 30-40 s from the first AP. Otherwise the sensory system will be reset (MacFarlane JM., 1892, Contr. Bot. Lab. Univ. Pennsylvania I; Hedrich and Neher, 2018; Nature Plants).

Moreover, the authors have not considered several important early publications, which contain detailed results relevant to the issue in question:

- Macfarlane, J. M. (1902). Contributions to the history of Dionaea muscipula Ellis. Contr. Bot. Lab. Univ. Pennsylvania I, 1, 7-44.
- Brown, W. H. and Sharp, L. W. (1910). The closing response in Dionaea. Bot. Gaz. 49, 290-302.
- Brown, W.H. (1916) The mechanism of movement and the duration of the effect of stimulation in the leaves of Dionaea. Am. J. Bot. 3, 68-90

The 3 independent studies mentioned above, conducted extensive series of repeated trigger hair stimulations, with varying pauses between them. The different authors agreed that at least two mechanical stimuli are necessary to produce closure of Dionaea leaves. It was found that trap closure can occur for consecutive trigger hair stimulations even when these were separated by pauses of 2 min or longer (although the number of stimuli required for trap closure increased as the time period between successive stimuli was expanded). This means that the 2-min recovery time used in this study was clearly insufficient for resetting the sensory system. Hence, what is interpreted as trap closure after one deflection might actually represent the result of the cumulation of consecutive subthreshold trigger hair stimulations that finally pass the threshold.

We thank Reviewer 2 for this comment. Indeed, we forgot to explain the 2 min dwell time between the experiments. We were well aware of these publications as they served us to find a reasonable waiting time between two consecutive (independent) stimuli, which allowed a system reset as well as efficient measurements. As stated in all the three publications, with a gap of 2 min between stimuli at least five stimuli (average 6x) were necessary to trigger a reaction at temperatures below 30 °C. Moreover, the closure after repeated stimulation was incomplete and/or slow. We performed our experiments at room temperature (19-21 °C) and took only those traps into account that closed rapidly (see Video S3). For the majority of our data points (25 out of 31), we repeated the deflection less than 5x before the traps eventually closed (on average 3.4x for all 31 samples).

Another hint indicating that the 2 min waiting time was sufficient becomes evident from the fast double deflection experiments (Fig. 2). If 2 minutes were not enough to reset the system, we should have observed closure (and the associated AP) coinciding with the first deflection after the 2 min wait, which never happened.

For the reasons mentioned above, we are convinced that our data are valid and not an artifact of a summation effect. However, we thank Reviewer 2 for making us aware that we should explain our reasoning for the 2 min gap choice. We added the explanation to the text, including the references mentioned by Reviewer 2 (refs. 23-25 in the revised manuscript). The temperature regime during the experiments is now mentioned in the Materials and Methods.

Authors: Then the procedure was repeated with increasing angular displacements until the snapping mechanism was triggered (Fig 1C and 1D), which was the case when a median displacement threshold of = 0.18 rad or a median torque threshold of = 0.8 μNm (n=21) was reached (Fig 1E and S1A Fig). Therefore, an animal has to apply twice a force F around 0.5mN close to the tip of the sensory hair, or up to 5mN close to the constriction (S1B Fig) to trigger closure.

Question: The same question as before: are these numbers relevant to the trigger hair-prey interactions observed in real life? Are these forces in a physiological range?

The sensitivity reported here is lower than that recorded in ref 18 (mean thresholds for hair displacement in large traps: 0.078 rad and torque: 159.5 nNm).

Could the result the authors have recorded, result from rather artificial experimental protocol conditions, in particular high angular velocities? (what were the actual angular velocities for these tests?). Maybe higher forces are found because of an unfixed trap. If stimulation moves the entire trap as well as the sensory hair, inaccurate values will result.

In contrast to later steps (formation of the “green stomach,” i.e. secretion), snap triggering is a purely (electro-)mechanical affair (at least according to the literature). Therefore, it does not matter whether the sensory hair is deflected by a prey animal or by inorganic matter, and the numbers presented in our manuscript are indeed relevant. As mentioned before, we tested the range of the trap’s operability, which cannot be done with living animals. Of course, these forces are in the physiological range since the sensory hairs are obviously deflected.

About the differences in sensitivity between our results and those of Scherzer and colleagues, we can only speculate. In the Supplementary Video S4 of Scherzer et al., the sensory hair hardly bends at the constriction but rather deforms at the lower part of the lever (please compare with our video sideview_bend.avi and new Video S2 in the revised manuscript). In our experiments, we observed this effect only on dehydrated leaves, which we did not use for measurements for this very reason. Therefore, contrary to Reviewer 2’s assumption, one reason for the differences in sensitivity could be that we measured traps in a natural, healthy condition, while the specimen from Scherzer et al. were perhaps starting to dehydrate as they were bisected. Alternatively, it could be a problem of sensor calibration. Again, we trust our system more because the MEMS sensors are calibrated by the Swiss Federal Office of Metrology (METAS), while Scherzer and colleagues did not calibrate their sensors via a certified institution. Fig. S1 F of Scherzer et al. shows that in the region of interest (0.16 µNm, 4.5°) their sensor is rather inaccurate.

The angular velocities used in our experiments are shown in Fig. 1 D and Fig. S1 A, respectively.

The traps did not move during the experiments (see new Video S2 in the revised manuscript and sideview_bend.avi).

Authors: When the two consecutive deflections were well below the displacement threshold, we never observed an AP. For deflection amplitudes near the displacement threshold, a single AP was elicited after the second deflection. This indicated that both deflections contribute to the RP, and that the AP induction threshold was only reached with the second deflection.

Question: Without having measured the membrane potential of the mechanosensitive AP-generating TH cells, the authors are not allowed to come to conclusions about electrical phenomena in the actual trigger hair. Thus, statements remain pure speculations.

Our statement is based on the well-established fact that RPs are transient potentials whose amplitude depends on the magnitude of the stimulus, and which can trigger APs when reaching a certain threshold. There was some excellent work done in the Venus flytrap sensory hairs showing the relationship between RP and AP in the specific case we are describing here (see Jacobson, 1965, J Gen Physiol.; Benolken, 1970, J Gen Physiol). We find it astonishing that elsewhere Reviewer 2 questions the usefulness of repeating an experiment that has already been carried out but in this case criticizes that we rely on experiments that have been conducted earlier to keep our characterization non-invasive.

Authors: .. we deflected the sensory hairs far beyond the angular displacement threshold and kept that position for 30 s (Fig 1F).

Comment: This situation is very artificial. What should readers learn from the outcome of the experiment about the biology of trigger hair-prey interactions?

In our work, we study how the electromechanical mechanism of sensing in the sensory hair leading to trap closure works. It is crucial to apply different, precisely controlled stimuli onto the hair while recording the response to investigate how the hair works as a sensor. This experiment clearly shows that not the stimulus (angular displacement), but the rate of the stimulus (change of angular displacement) is sensed by the sensory hair of the Venus flytrap.

Authors: If sustained displacement had contributed to the RP, it should have remained above the threshold, in which case we would expect a series of APs.

Comment: This is pure speculation again, because the authors have used surface potential electrodes only rather than placing a sharp voltage recording microelectrode in the trigger hair bending zone (Benolken et al. 1970 and Hodick, Sievers. 1988) and thus have no clue about triggered membrane potential changes in the mechanosensor. Therefore the statement “.. the deflection rate plays a pivotal role to build up the RP while static deflections do not contribute” is too far-fetched and in stark contrast to previous work (Benolken et al., 1970; Volkov et al., 2008). Moreover, the ECB model would benefit from high-resolution membrane potential recordings able to resolve the charge increment ∆Q and the RP and thus the threshold for closure.

We have indeed not measured the RP directly and relied on previous work. However, rather than speculation, we think these statements are based on well-founded assumptions, for reasons we have explained above. Benolken confirms our statement (see Fig. 4 in Benolken, 1970, J Gen Physiol). As we state in the manuscript, we think that Volkov’s results for extended stimuli are an artifact of his manual deflection method and that, in fact, multiple stimuli occurred due to shaking. This problem has been recognized a while ago (see Brown WH, 1910, Bot Gaz). We have not observed a single case of multiple APs or trap closure upon sustained displacement.

Authors: Based on these findings, we developed a simple model to explore the limits of angular displacement and velocity within which the traps would react. In our electromechanical charge buildup model, a mechanical deflection leads to a charge buildup of the RP ..

Comment: What is the value of a model on the behaviour of the RP without having measured it?

All the authors did is measure the trap surface potential that is no more nor less than a qualitative proxy of the AP generated in the TH.

We agree with Reviewer 2 that RPs can be measured, however only invasively. In our studies, this was not necessary because there is information in the literature and our model, in combination with our experimental data, confirms these previous findings.

In general, a model is needed if not all the parameters are known or if they cannot be directly measured (e.g. due to invasive nature of certain measurements).

Authors: If the accumulated charges surpass a certain threshold value, an AP is elicited.

Comment: This fact is neither in question nor new, but was already documented by papers from the Volkov lab before. By the way, induction of AP in response to charges accumulated that pass a certain threshold is not a trigger hair-specific feature but applies to, for example, the midrib as well (Volkov et al., 2008).

We never claimed that this is new. We agree with the second statement as it is well known that the entire trap is excitable. However, in our manuscript, we do not discuss the midrib but focus on the sensory hair of the Venus flytrap, which is the primary sensor and acts as an amplifier of mechanical signals, rendering the trap very sensitive.

Authors: .. we observed trap closure induced by single deflections at lower angular velocities. To narrow down the range in which this occurs, we repeatedly deflected the same sensory hair with varying angular velocities until the trap closed.

Comment: This experimental scenario again was not linked to any kind of trigger hair-prey collision observed in vivo. What does the reader learn from the results obtained? How many action potentials were triggered by these single deflections? Only two, such as in experiments shown in Fig 3C/D? Were 2 APs always triggered before the trap closed? This information should be provided for all experiments.

We answered this point already several times. The work of Scherzer and colleagues is, to our knowledge, the only case studying the catching mechanism with living prey (but bisected traps). Does the reviewer suggest that all the previous work done on the snapping mechanism is useless because the experiments have not been performed with prey animals as triggers?

We thank Reviewer 2 for her/his question about the number of APs in single deflection experiments. It always needs two APs for trap closure, which is indirectly mentioned in the text but, as we realized now, not explicitly enough. We changed the text to make this clearer.

Authors: Between two consecutive deflections, we waited 2 min for the trap to recover and any RP to dissipate.

Comment: (see comment above)

This is again pure speculation, because the authors have not measured the TH RP.

Our response to this comment can be found above.

Authors: .. the closing torque around the midrib close with a median of 0.65mNm is a better quantity to characterize the trap’s closing force (Fig 4C). The delay time, i.e., the time between the mechanical stimulus and the start of trap closure, was 0.6 0.3 s (mean s.d., n = 18), which is in the same range as the 0.4 s previously reported [25].

Comment: The time resolution of the force sensor is probably not too well suited to mechanical stimulus and the start of trap closure. Why not compare delay times with those obtained with non-invasive optical methods?

We are glad that Reviewer 2 points this out. In fact, we used optical methods but failed to mention it in the text. Time resolution of the snap force sensor (readout frequency 10 Hz) would have been good enough but we did not use it for a lack of synchronization with the camera. We now specify this in the text and added an explanation in the Materials and Methods section.

Authors: In a natural situation, when the deflection is fast and the angular displacement large enough, i.e. when an insect touches the sensory hair, the RP threshold is reached and a single AP is generated. In the case of a slower deflection, the RP threshold is reached during the deformation, eliciting an AP while the membrane is still under strain, thus reaching a second RP threshold during the same deflection. A second AP and trap closure are the consequence. Since the velocity range for single-touch snapping is far below that of the classical Venus flytrap prey, we can only speculate about the relevance of this observation in nature.

Comment: In the Discussion the authors themselves conclude that their experimental settings were non-natural. So why should the reader bother about a situation that is not natural?

As previously mentioned, our goal was to describe the Venus flytrap system under all possible situations from an electromechanical perspective. The single-trigger snapping is a fact that was predicted by our model and experimentally confirmed. Therefore, a slow, single deflection is one possible scenario to which the Venus flytrap can react with trap closure. About its role in nature we can unfortunately only speculate. Thanks to Reviewer 2 we realized that the word “natural” is somewhat unfortunate in this context. We changed “natural” in line 151 to “classical”.

In addition, we would like to highlight that the broader scientific audience of PLoS Biology is not limited to the Venus flytrap community but might also be interested in the general functionality of and observations regarding sensory cells in plants.

Authors: … either a maximum force F or a maximum linear displacement d is set to define the maximum deflection (Fig 6D).

Comment: For which experiments was force feedback control used? No data appear to be shown in the paper. What was the angular velocity in this case?

We used force feedback for the single deflection experiments shown in Fig. 3. The maximal force was chosen to get maximal sensory hair displacement without damaging the sensor. For the fast double deflections, we set a maximal linear displacement (Fig. 1) because we wanted to incrementally increase the angular displacement. The corresponding angular velocities are shown in Fig. 3 B. We added an explanation to the Materials and Methods section.

Authors: Video S2.

Comment: It is not clear how angular deflection was extracted from this experiment. First, it appears that the force transducer and the direction in which it moves are not perpendicular to the axis of the hair. In the beginning of the deflection, the sensor can be seen moving to the left (and probably downward), deflecting the trigger hair to the left, but at 11 sec, the trigger hair snaps off the sensor tip, resulting in a movement further to the left (and probably upward).

Video S2 is now Video S3 in the revised manuscript. Displacement is perpendicular to the hair. The optical alignment is not perfectly parallel to the line of the deflection sensor movement. We measure deflection only until the point of slipping, which occurs because of trap closure.

Second, the deflection is obviously influenced by the closing movement of the trap but this is not reflected in the angular displacement trace in Fig.3C.

The motion artefacts are indicated in Fig. 3 C and D by dashed lines. We adjusted the caption to make this clearer.

Third, the point of force application seems to move slightly towards the hair tip during the experiment.

Unfortunately, this is very difficult to avoid for a linear displacement of a bending specimen. Therefore, we mention the torque at the hair’s base, which is not affected by this.

Fourth, the video is not fully synchronised with the force trace (the trap movement at 3.8 sec can be seen in the video before the change in the force).

We are showing the force of the deflection sensor (not of the snap force sensor). We made that clearer in the caption.

Authors: Figure 3A,B

Comment: It seems that amplitude and angular velocity were varied simultaneously in these experiments, making it difficult to study the effects of one particular factor.

We only varied the angular velocity. The impression that the angular displacement changes is caused by the overshooting of the sensor due to high velocity. In other cases with fast deflection, the maximal force was reached with a smaller displacement as the hairs appear stiffer at higher velocities (viscoelastic effects). Since such effects are indeed difficult to represent, we chose to present our data in phase diagrams.

Authors: Fig 1C - 2 consequent touches were carried out within one second, and the trap closure was examined.

Comment: I wonder about the high frequency of stimulation. Since the AP lasts over one second (see Fig 1 C 193 s) is it ok to stimulate faster? Ref 18 reported desensitization at frequencies above 0.25Hz (one touch every 4 s). What is the physiological stimulation frequency?

Desensitization at 0.25 Hz occurs after more than 4 stimuli. We do not know what happens in terms of desensitization after two stimuli with a 1 s interval. However, MacFarlane published in 1892 that an interval of 0.33 s leads to trap closure while an interval of 0.25 s does not (MacFarlane JM, 1892, Contr. Bot. Lab. Univ. Pennsylvania I). Since an AP is an all-or-nothing response, we think it is okay to stimulate at the chosen frequency.

Authors: line 67: Volkov Ref 20

Comment: this is supposed to be #23.

We thank Reviewer 2 for spotting this mistake. We corrected it (in the revised manuscript this is now ref. 26).

- line 97: ´the higher boundary was`…

Comment: Where are the data shown? Is the setup able to precisely perform such quick and far movements?

Yes, the setup is able to perform these movements. However, given the higher velocity, the number of data points is reduced. Nevertheless, force feedback control still works. All data for the different boundaries are shown in Fig. 3 B.

- line 98: ´Additionally, we performed another set…`

Comment: Where can the reader find the data?

The data set is presented in Fig. 3 B.

Conclusion:

Most of the findings the authors present are not new.

To our knowledge, the single deflection trigger is new and in our view very interesting even if we do not know what exactly it means for the biology of trap-prey interactions. Measuring the mechanical basis of the trap closure mechanism on intact Venus flytraps in combination with the measurement of the APs is also new. Additionally, we provide a new model that integrates both mechanical and electrical effects. Therefore, even if Scherzer and colleagues, who must have done the experiments largely in parallel to us, published their findings before us, we are convinced that our manuscript contains enough novel results and will be very interesting for the readers of PLOS Biology.

Confirmatory results are, however, not discussed in the context of previous findings by other labs.

The experiments performed to support a model scenario of one strike gaining 2 APs appear ill defined, and artefacts resulting from insufficient pauses between stimulations, and interference from the snap force measurement have not been considered.

We think that in science the value of a given finding increases dramatically if it is independently confirmed by others. Although it is not correct that we have not discussed the findings by other labs, we have extended the discussion in the revised manuscript.

The authors concluding about the RP of the trigger hair AP generating cells without measuring the membrane potential is a No-Go in bioelectrics.

We do not agree with the reviewer’s comment because we did not make any quantitative statements about the RP. But from our readouts and previous findings, we can infer what happened with the RP, eventually leading to APs and trap closure.
