# Supplementary material for: A single touch can provide sufficient mechanical stimulation to trigger Venus flytrap closure
Source: PLoS Biol. 2020 Jul 10;18(7):e3000740. doi: 10.1371/journal.pbio.3000740 (PMC7351144; doi:10.1371/journal.pbio.3000740)
Supplement: Attachment — Submitted filename: ResponseToReviewers_2.docx [file pbio.3000740.s009.docx]

## Detailed Response to the Reviewers’ Comments 2

### Reviewer 1

The authors have addressed most of my concerns. Additional points about the response:

“Using angles allows us to normalize for different contact heights and different sensory hair geometries. We have good evidence that the sensory hair bends mainly at the constriction, whereas the shaft is hardly deformed.” This needs to be stated explicitly in the manuscript.

We agree with Reviewer 1 and added a statement in the manuscript (line 44).

“Using angles allows us to normalize for different contact heights and different sensory hair geometries. We have good evidence that the sensory hair bends mainly at the constriction, whereas the shaft is hardly deformed.” Please include this as a brief discussion point.

We added that to the discussion (line 186).

### Reviewer 2

The sensory biology of plants in general and carnivorous plants in detail will attract the broad readership of PlosBiology.

Authors rebuttal: Our mechanical data are largely in agreement with those recently published by Scherzer and colleagues, .. Comment: The unexpected new finding of the authors is that a trigger hair of Dionaea has to be touched only once to close the snap trap.

The author’s observation, however, not only “contradict the old dogma that two deflections are necessary to provoke trap closure” but also the very similar study that was published in Nature Plants recently. The reader of the PlosBiology paper thus expects the finding of “one strike is enough” to be sufficiently supported by experimental evidence. In response to the initial version, this referee asked - because of the lack of experimental data - for the authors to tone down assumptions about the MS channels and behaviour of the resting potential (RP). Furthermore, the authors were asked to increase the delay in stimulation to more than just 2 min and in an independent test series use traps that were not stimulated before. The authors, however, have ignored these reasonable suggestions completely. Since the authors’ studies were not submitted to a journal specialising in biomechanics/biophysics but PlosBiology, the reader of a biology journal needs to see the new “one touch is enough” finding discussed in a biological and evolutionary context.

We thank Reviewer 2 once again for the important points he/she made, which greatly helped us to improve the quality of our manuscript. However, we think that the similarities between our manuscript and the one published in Nature Plants are limited to the mechanical parameters of the trigger hairs, which are largely in agreement. Concerning the range of possible parameters, our study goes far beyond what has been published in Nature Plants and, indeed, we present the novel insight that under certain conditions one deflection is sufficient to trigger the closure mechanism. We do not understand the reproach of exaggerating our assumptions about electrophysiological events that occur between the mechanical stimulus and the triggering of an action potential (AP), since we clearly state in the manuscript that these are assumptions based on the available literature. In our extensive response to the previous comments of Reviewer 2, we explained in detail, also based on literature suggested by Reviewer 2, why we consider a two-minute delay between successive measurements on the same trap to be appropriate. The suggestion to perform “single deflection” experiments on plants that had not been stimulated before is reasonable but, contrary to what Reviewer 2 states now, has not been made in his/her previous comments. However, we do have data where a single deflection triggered trap closure without pre-stimulation. They were not included in the “single deflection” dataset used for Figure 3 B because this experiment was designed to determine the parameter boundaries, i.e. moving from a set of parameters that did not result in trap closure to the parameter space where trap closure occurred. We specify this in the second revision of the manuscript and present the plots showing single deflection trap closure without pre-stimulation in Supplementary Figure S3. Furthermore, Supplementary Video 2 shows such a case. We adjusted the caption to clearly point this out.

Detailed comments and questions: Title: 1. Comment: To get informed about the new findings right away, the reader should learn already from the title that mechanical and electrical properties of the trigger hair allow the trap to close after one stimulation

We changed the title according to Reviewer 2's suggestion to "A single touch can be enough: quantitative measurements of mechanical parameters shed light on the snapping mechanism of the Venus flytrap."

Abstract: Authors: Our results confirm the model’s predictions, suggesting that the Venus flytrap may be adapted to a wider range of prey movement than previously assumed.

2. Comment: The readers have fund situations where the trap closes after one stimulation. Is 1 the “wider range” to 2? Does the model propose situations where more than 2 touches would be required to close the trap? In this context it should be mentioned that in their PNAS paper Escalante-Pérez et al. (2011) showed Dionaea under water stress required more than 2 touch APs to close the trap.

From the text in the main part of the manuscript it becomes clear what we mean by “a wider range of prey movement”, namely an extension of the prey’s speed towards slower movements. However, as Reviewer 2 cunningly admonishes, the model indeed predicts situations, in which more than two deflections would be necessary, as one can easily see in Figure 2 B. In this context, we observed situations where more than one deflection was necessary to elicit an AP (e.g., Figure 1 C, middle graph), so more than two deflections would be necessary to close the trap. This shows that sub-threshold stimuli can be added up (if delivered close together) to reach the RP threshold. We added a comment in the text (line 110).

In the case described by Escalante-Pérez and colleagues a decrease in responsiveness induced by water stress led to an increase in the number of deflections necessary to trigger trap closure. Unfortunately, the authors did not measure the APs in the water stress experiments. Therefore, the comment of Reviewer 2 is speculative. However, if we assume that in those experiments each deflection in water-stressed plants lead to an AP, more than two APs were necessary to reach the threshold for trap closure. This is different from the situation in our model based on well-watered and fully responsive plants, where two APs are always sufficient to reach the threshold in the “short term memory (STM)” (if elicited less than 30 s apart). To simulate the situation induced by water stress in our model, we would either have to increase the STM threshold or reduce the charge transmitted by an AP, so that more than two APs are necessary to close the trap. This would be tantamount to making the trap less sensitive. Again, since we studied healthy, well-watered plants where two APs are always sufficient to initiate trap closure, the findings reported by Escalante-Pérez et al. (2011) are not relevant in our context.

Introduction: Authors: Although there is a general agreement that sensory hair deflection opens mechanosensitive ion channels, such channels have not yet been identified [17, 18].

3. Comment: In Arabidopsis and genomes of other plants as well MSLs and OSCAs have been identified as mechanosensitive channels of different force sensitivity and ion (Cl- vs Ca2+) selectivity. Without knowing which MS channels of which properties are expressed in the trigger hair of Dionaea, how could the authors derive a proper model on touch induced APs?

We fully agree with Reviewer 2 that mechanosensitive channels have been shown or proposed in Arabidopsis and other species. However, we are talking about the sensory hair of Dionaea, which, given the context of the paper, does not need to be specified in our opinion. In our electromechanical charge buildup model, we use charge (a general change in membrane polarization), for which the exact nature of the causing ions, and thus the channel transporting them, is irrelevant.

Authors: Previous attempts to correlate the mechanical stimuli to the generation of APs suffered from the lack of appropriate instrumentation [10, 19] and thus were not quantitative, or the experiments were done in vitro using dissected traps [20]. To overcome these shortcomings, we used a microelectromechanical (MEMS)-based force sensor mounted on a microrobotics system to precisely control the velocity and amplitude of the deflection and to simultaneously measure the applied force in vivo (Fig 1A and 1B, and 5). .. and In addition, using a non-invasive method, we measured APs to test the deflection conditions under which they are generated.

4. Comment: this statement is wrong! Ref. 20 had used a “system to precisely control the velocity and amplitude of the deflection and to simultaneously measure the applied force in vivo”. Cutting of one lobe from the trap on an otherwise intact plant the authors cannot classify “in vitro”. The reader also should not be given the wrong impression that Jacobson and Benolken [19] having performed with excised (ex planta) trigger hairs represent in vivo (in planta) studies.

We agree with Reviewer 2 that Scherzer and colleagues used a system capable of performing controlled deflections and measuring the resulting force. However, as we already stated in the previous response letter, they unfortunately do neither mention nor show the state of the traps/plants they used for the mechanical measurements. Supplementary Video 4 in their paper shows one lobe of a trap that is unnaturally twisted and certainly not in a natural, undisturbed state. Reviewer 2 has the point that it is questionable whether this can be called in vitro, since a trap in such a state is probably still alive, though hardly capable of catching prey. To avoid the impression that Jacobsen and Benolken performed their experiments in vivo and to circumvent the in vitro ambiguity, we changed the text to “… not quantitative, and/or the experiments were done in fixated or dissected, non-functional traps [20].” (Line 24)

Results: 5. Receptor potential: The paper is so imprecisely written in many places that the general reader and even reviewer #1 believe that receptor potentials are measured after all. When mentioning receptor potential in Results, the authors should clearly state that they in their study did not measure receptor potential features but assume it. Without measuring RPs under their experimental conditions, the authors have to make clear what is fact and what is speculation when talking about RPs.

Neither in the first nor in the second round of Reviewer 1’s comments could we find any indication that he/she believes that we have measured RPs. Furthermore, we clearly describe what we measured, namely APs, and how it was done. To comply with the wish of Reviewer 2, however, we inserted the following text in the revised manuscript: “Since RP measurements are destructive and not compatible with our non-invasive in vivo experiments, we relied on data from the literature [10, 19].” (Line54)

Authors: These results suggest that a fast deflection of the sensory hair increases the RP to a certain level, which depends on the amplitude of the angular deflection. RPs can add up and may elicit an AP after several deflections if they are below the deflection threshold.

6. Comment: How come the authors think that a voltage drop of certain amplitude suffices to initiate an AP in the trigger hair podium? In plants, electrical signals are associated with cytoplasmic Ca2+ signals. In the giant alga Chara system, mechanical stimulation elicits RPs as well as APs, after 50 years of research it is not yet clear how mechanistically the RP translates into an AP but clearly shows that cytoplasmic Ca2+ is a key player (Shepherd et al. 2008 and papers cited).

We are not sure what Reviewer 2 wants to say here. The molecular and mechanistic base of how RPs are translated into APs is irrelevant for our model. Therefore, we don’t speculate about it. What matters is the change in membrane polarization based on charge relocation, which is very well described in Benolken and Jacobson, 1970, for example, where they showed hat every AP is preceded by an RP, which is a voltage drop (change) across the membrane. A schematic representation can also be found in Figure 5 of our manuscript.

Authors: Although a summation effect was reported in these publications, at least five deflections spaced by 2 min were necessary to induce trap movements, which were always slow and/or partial [23-25]. After the dwell time, the procedure was repeated with increasing angular displacements until the snapping mechanism was triggered (Fig 1C and 1D).

7. Comment: Using traps without a pre-stimulation history the authors should show that the trap can close on one applying an above critical angular displacement. The result of this experiment will support or falsify the authors prediction and thus is a must. At the IPBC congress a poster (provided to the PlosBiology editor) was presented showing that Dionaea plants were generated that express the genetically encoded cytoplasmic Ca2+ sensor. With a single touch the Ca2+ in the bending zone of trigger hair podium rises but stays blow the threshold required for trap closure. Upon the 2nd touch Ca2+ level passes the threshold and the trap closes. The authors have not observed that a single touch causes Ca2+ to rise to an extent that is able to close the trap.

As detailed in our response to the introduction of Reviewer 2’s comments, we now present data where a single deflection on traps that were not pre-stimulated leads to trap closure (Supplementary Video 2 and Supplementary Figure S3). Unfortunately, we do not have such Venus flytrap plants expressing a Ca²⁺ sensor that Reviewer 2 refers to. Therefore, it was not possible for us to monitor Ca²⁺ levels during our experiments. We agree that this would be a nice tool to test our findings, even if it is not clear at which stage of the electromechanical events Ca²⁺ plays a role.

Discussion: Authors: We performed all of our experiments on intact plants. In our hands, detachment or bisection of the traps quickly led to partial dehydration and lower forces were necessary to deflect the sensory hair.

8. Comment: Such statement without having presented data is a no-go in science. Authors are asked to describe in detail the experimental procedure, documenting what the authors call “partial dehydration” and showing statistically significant results. From the information provided, interested readers must be able to repeat the experiment and confirm the findings published in Plos Biology. When showing and discussing the so far “not shown data” the authors may explain to the reader how partial dehydration could increase the mechanical sensitivity of the trigger hair. Why? In the PNAS paper Escalante-Pérez et al. (2011) observed the opposite phenomenon. When Dionaea suffers from drought “partial dehydration” the capacity for trap closure is reduced. In Forterre et al. Nature (2005) even excised traps were well suited kinematic and mechanical measurements that provided the basis to model the biomechanics of trap closure. So far there was no study showing that intact traps on the plant and those with one lobe removed respond differentially to mechanical stimulation.

We precisely described our experiments to allow interested readers to repeat them. To make it even more clear, we added “well-watered” to the sentence in question, a term which is also used by Escalante-Pérez and colleagues (Escalante-Pérez et al., 2011). The sentence criticized by Reviewer 2 discusses the observed, slight differences in the sensitivity of the sensory hair between the work of Scherzer and colleagues, and ours. Since we are only presenting data collected from intact, well-watered plants, we do not think that it is necessary to discuss the influence of partial dehydration in detail.

In our view, Reviewer 2 mixes two different things. What we compare in this sentence is the sensitivity of the sensory hair to trigger an AP, while Escalante-Pérez and colleagues looked at the capacity for trap closure. While two APs are indeed necessary to trigger the snapping mechanism, the latter does not seem to work well in a (partially) dehydrated state. This is not really surprising, since the final snapping is assumed to be a turgor-controlled process and, therefore, depends on adequate hydration. This is in agreement with our own observations, where dehydrated traps did not close at all or only very slowly and incompletely although APs were fired. Therefore, we did not use these results for our analysis.

Authors: Since the velocity range for single-touch snapping is far below that of the classical Venus flytrap prey, we can only speculate about the relevance of this observation in nature. However, one can imagine that it could be an advantage for catching slower prey animals, such as slugs, snails or larvae, which may not touch a hair twice within a 30 s time span but for which anecdotal evidence suggests can be prey.

9. Comment: this statement cannot go without proper citation. How often if at all have slugs, snails or larvae been found in closed flytraps in their natural environments? YouTube videos showing all kinds of small animals could be offered as meal for Dionaea, is not of any biological relevance.

That’s why we call these observations anecdotal and clearly state that we can only speculate.
